# Long-Term Durability of Ciliary Neurotrophic Factor–Releasing Revakinagene Taroretcel-lwey in Individuals With Retinal Degenerative Disorders

**DOI:** 10.1167/iovs.66.11.3

**Published:** 2025-08-04

**Authors:** Konrad Kauper, Arne Nystuen, Lisa Orecchio, Eugene Gonzalez-Lopez, Alice Lee, Jacque L. Duncan, Jay M. Stewart, Thomas Aaberg

**Affiliations:** 1 Neurotech Pharmaceuticals, Inc., Cumberland, Rhode Island, United States; 2 University of California San Francisco, San Francisco, California, United States; 3 Retina Specialists of Michigan, Foundation for Vision Research, Grand Rapids, Michigan, United States

**Keywords:** cell therapy, ciliary neurotrophic factor, encapsulated cell technology, intraocular administration, neuroprotection, retrospective analysis, retinal degenerative disease, revakinagene taroretcel-lwey

## Abstract

**Purpose:**

This retrospective analysis examined drug release levels and long-term function of revakinagene taroretcel-lwey (formerly known as NT-501), a ciliary neurotrophic factor (CNTF)‒releasing encapsulated cell therapy, following implant durations of 0.5 to 14.5 years in participants with retinal degenerative disease.

**Methods:**

Explant samples were collected from participants in 6 clinical trials (NCT00063765, NCT00447993, NCT00447980, NCT01530659, NCT03319849, and NCT00447954) and assessed for release rate of CNTF, histomorphology of encapsulated cells, and CNTF integrity and activity. Serum samples were tested for CNTF levels as well as antibodies to CNTF and the CNTF-producing NTC-201-6A cells.

**Results:**

A total of 49 explanted revakinagene taroretcel-lwey devices were analyzed. Analyses showed that they produced steady levels of bioactive CNTF over a duration of 14.5 years. The weighted mean rate of CNTF produced averaged 1.6 ng/day (95% confidence interval; [CI] = 1.388 to 1.840), a rate shown to be effective in slowing photoreceptor loss. Potency of secreted CNTF was tested from a subset of explants and showed that CNTF from explanted devices remained equally bioactive compared with a CNTF reference standard. Explanted devices produced CNTF glycoforms at the expected molecular weights. Histological evaluation comparing preimplant cells to those explanted over 14.5 years revealed cells were similar in distribution and morphology. There was no evidence of an immunological reaction to CNTF or the NTC-201-6A cells.

**Conclusions:**

This retrospective analysis suggests that a single, intraocular administration of revakinagene taroretcel-lwey provides sustained, bioactive CNTF for durations exceeding a decade, potentially providing a long-term treatment option for chronic retinal degenerative diseases.

Retinal degenerative diseases, a heterogeneous group of chronic conditions, are a significant cause of vision loss worldwide and have a profound impact on an individual's quality of life.^1^^,^^2^ The hallmark of these diseases is damage to the retina, which leads to progressive vision loss, marked by damage to both the photoreceptors and the retinal pigment epithelial cells.^1^^,^^2^ Few effective treatments are available.

Müller glial cells are essential to the health and integrity of photoreceptors by providing both structural and cytokine support.^3^^–^^5^ One cytokine, ciliary neurotrophic factor (CNTF), is key in stimulating Müller glial cells to produce the various neuroprotective factors critical for preserving retinal neurons.^3^^,^^5^^,^^6^ Under pathological conditions, neurotrophic factors, including CNTF, are released by Müller glial cells.^5^ CNTF binding to its receptor activates the Janus kinase/signal transducer and activator of transcription survival pathway, shifting Müller glial cells to a neuroprotective phenotype and enhancing the survival of photoreceptors.^4^^–^^6^ Consequently, CNTF has the potential to promote the survival of retinal cells in retinal degenerative diseases, such as retinitis pigmentosa (RP), age-related macular degeneration with geographic atrophy, glaucoma, and macular telangiectasia type 2 (MacTel).^6^^–^^10^ This has been borne out in preclinical studies, which have demonstrated that sustained treatment with CNTF offers long-term protection of photoreceptors.^3^^,^^11^^–^^13^ The levels of CNTF in the vitreous of patients and controls have not been determined for the retinal degenerations in these studies; therefore, it is not possible at this point to state whether supplying exogenous CNTF would be supplementing a deficiency or a disease-modifying therapy that is agnostic to molecular pathology. Despite this potential, the relatively short half-life of CNTF limits its therapeutic potential when administered through traditional methods, such as intravitreal injection.^14^

Revakinagene taroretcel-lwey (formerly known as NT-501; Neurotech Pharmaceuticals, Inc., Cumberland, RI, USA) is an intraocular encapsulated cell therapy (ECT) that contains the human retinal pigment epithelial cell line NTC-201-6A, which expresses recombinant human CNTF (rhCNTF). Revakinagene taroretcel-lwey is implanted into the vitreous cavity, delivering CNTF into the vitreous humor, thereby overcoming the limitations of a short half-life and need for frequent injections. The semipermeable membrane surrounding the cells and internal scaffold (Fig. 1) allows for the exchange of nutrients and oxygen necessary for long-term cell survival and acts as a barrier to the host immune system, lowering the risk of host rejection and intraocular inflammation.^8^^,^^15^

**Figure 1.**
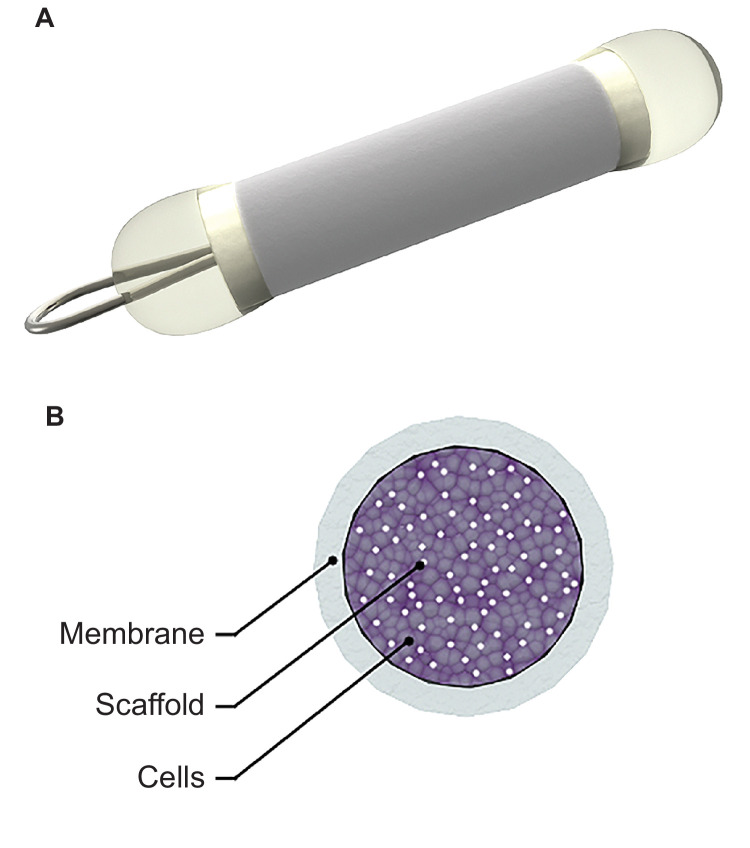
(**A**) Schematic of the revakinagene taroretcel-lwey ECT device. The revakinagene taroretcel-lwey ECT implant consists of a sealed, semipermeable, hollow-fiber membrane capsule surrounding a scaffold of polyethylene terephthalate yarn loaded with ciliary neurotrophic factor–secreting NTC-201-6A cells. A titanium suture anchor allows the device to be secured below the sclera. (**B**) Cells are encapsulated in an immunoprotective membrane that allows oxygen and nutrients to diffuse into the ECT device and therapeutic factors to be released into the vitreous. ECT, encapsulated cell therapy.

In preclinical models and clinical trials, revakinagene taroretcel-lwey has demonstrated in vivo delivery rates of approximately 1.6 ng/day of CNTF,^14^ which has been shown to be sufficient for slowing photoreceptor degeneration in retinal degenerative diseases, including RP and MacTel.^7^^,^^16^^,^^17^ Given the chronic nature of many retinal diseases, long-term treatment is essential. The stability of CNTF release from revakinagene taroretcel-lwey over 2 years of intraocular implantation in humans has been previously reported.^14^ The NTC-201-6A cell line has been shown to be stable with robust vector maintenance and CNTF output (Plakkot S, et al. *Invest Ophthalmol Vis Sci* 2023;64. ARVO E-Abstract 3662),^18^ and the capsule was found to be stable in a forced degradation study (Selander L, et al. *Invest Ophthalmol Vis Sci* 2023;64. ARVO E-Abstract 3670). To further assess the durability of revakinagene taroretcel-lwey, this retrospective analysis evaluated CNTF release levels and long-term function from a subset of explanted devices from 6 clinical trials.

## Materials and Methods

### Study Design

This retrospective analysis assessed six clinical trials conducted in participants with RP (NCT00063765,^19^ NCT00447993,^20^ NCT00447980,^20^ and NCT01530659), MacTel (NCT03319849^17^), and geographic atrophy (NCT00447954 [Jaffe GJ, et al. *Invest Ophthalmol Vis Sci* 2011;52. ARVO Abstract 1651]). One device was from a compassionate-use protocol (Emory 201-CU01-RDD-2011). Detailed descriptions of the study methods and participant characteristics have been published elsewhere,^17^^,^^19^^,^^20^ except for one of the phase II trials of RP (NCT01530659), which remains unpublished. Detailed descriptions of the revakinagene taroretcel-lwey implant and explant procedures have been published.^14^^,^^16^^,^^20^ Over the course of the revakinagene taroretcel-lwey clinical trial program, a portion of the implanted devices were explanted and returned to Neurotech because of protocol requirements, participant request, or physician recommendation. Removal was mandatory at the conclusion of the phase I trial (NCT00063765) 6 months after implantation. In the remaining trials, participants could opt for removal after the end of the trial. The devices reported herein were surgically removed after a range of 6 months to 14.5 years after implantation. Details of the assessed devices are provided in Supplementary Table S1.

### Outcomes

#### Sampling of Explanted Devices

To measure CNTF release, the explanted devices were incubated in 1 mL of Endo-SFM medium (Gibco BRL, Gaithersburg, MD, USA) at 37°C in 5% CO_2_ and 90% humidity for 24 hours. These samples were analyzed by the methods described below for CNTF release, and a subset of these samples was analyzed for bioactivity and identity.

#### Evaluation of CNTF Release Kinetics

The rate of CNTF released by explanted devices was evaluated via a human CNTF enzyme-linked immunosorbent assay (ELISA) using Human CNTF Quantikine ELISA, Cat. number DNT00 (R&D Systems, Minneapolis, MN, USA).

#### Evaluation of CNTF Bioactivity and Identity

The human erythroleukemic cell line TF-1.CN5a.1 (ATCC CRL-2512), which proliferates in a dose-dependent manner in response to CNTF, was used for a bioactivity assay.^21^ Quantification of the response to CNTF was performed by measuring the reduction of the redox dye formazan in Cell Counting Kit 8 (CCK8; Dojindo Laboratories, Rockville, MD, USA). Because of the small quantity of CNTF found in the samples, a modified version of the assay was performed in which 3 independent dilutions targeted to the historical half-maximal effective concentration (EC_50_) of 0.06 ng/mL were used rather than a full dilution series. A full dilution series of positive control reference CNTF was included on the plate as a comparison of activity response, and a negative control of no added CNTF was assessed. Activity bioassays were performed in a 96-well plate using the inner 60 wells, leaving a moat of wells around the outer edge of the plate with 200 µL of RPMI-1640 media. A dilution series of CNTF from control and replicates of a single dilution from explant-conditioned media was made in 100 µL of RPMI-1640 + 0.1% bovine serum albumin. A total of 20,000 TF-1.CN5a.1 cells in 100 µL of RPMI + 10% fetal bovine serum were added to each well. The plates were incubated at 37°C, 90% humidity, and 5% CO_2_ for 3 days. After incubation, 20 µL of CCK8 was added to each well and incubated at 37°C, 90% humidity, and 5% CO_2_ for 3 hours. The plate was read at 450 nm on a SpectraMax (Molecular Devices, LLC, San Jose, CA, USA) plate reader using SoftMax Pro version 5.2 and a comparison of the response (absorbance) at the historical EC_50_ was made between the sample and the control, with the final reported value reported as explant optical density (OD)/reference OD.

#### Capillary-Based Immunodetection

Revakinagene taroretcel-lwey–derived CNTF, a unique glycosylated form of CNTF that migrates at 33 kDa, was detected by the ProteinSimple Jess capillary gel electrophoresis and automated simple Western blot system with chemiluminescence detection (Bio-Techne, Minneapolis, MN, USA). For gel electrophoresis, the samples were reduced and denatured at a final dilution of 1:2 in gel electrophoresis reagents. The 2 purified protein-positive controls were native rhCNTF (R&D Systems) made in *Escherichia coli* and rhCNTF purified from NTC-201-6A cells. Proteins were at 15 pg/µL by OD280 in gel electrophoresis reagents, reduced, and denatured prior to electrophoresis, which was performed on a ProteinSimple 12 kDa to 230 kDa separation cartridge (12‒230 SM-W004) with Standard EZ Pack 1 (PS-ST01EZ-8). Run parameters were 30 minutes, 2:1 stacking ratio, 18-second matrix, and 9-second sample. The Western blot primary antibody was rabbit monoclonal anti-CNTF (Abcam ab267716), and the secondary antibody was ProteinSimple anti-rabbit HRP Detection module (DM-006). Both primary and secondary antibodies were incubated for 30 minutes after a 5-minute blocking step. Exposure was high dynamic range.

#### CNTF Serum Levels and Immunologic Response

Participant serum samples were collected at baseline (preimplantation) and at 3, 6, and 12 months after implantation. Some participants also had samples collected at 24 and 30 months after implantation, per trial protocol. Serum CNTF levels were determined by a CNTF ELISA kit, as previously described.^14^ An ELISA was also used to quantify specific anti-human CNTF antibody titers after incubating participant serum on a plate coated with rhCNTF (R&D Systems). The signal was detected using a secondary antibody, horseradish peroxidase (HRP)-conjugated donkey anti-human immunoglobulin G (IgG; Jackson ImmunoResearch, West Grove, PA, USA). NTC-201-6A cell antibodies were determined using an ELISA by incubating the participant's serum on a plate coated with NTC-201-6A cells, followed by probing with HRP-conjugated donkey anti-human IgG (Jackson ImmunoResearch). For the antibody screening, a positive result was determined by the observation of increased titer from baseline by two five-fold dilutions or greater from the preimplant titer assayed on the same assay plate. The titer was defined as the largest dilution of serum that yielded an OD of 6 standard deviations of the mean backgrounds observed during method qualification: OD >0.084 for anti-rhCNTF and OD >0.130 for anti-NTC-201-6A.

#### Macroscopic and Histological Evaluation of Revakinagene Taroretcel-lwey

Macroscopic evaluation of explanted devices was conducted at 25 × magnification (Nikon Optizoom microscope, Tokyo, Japan) to document signs of damage or structural or physical changes. Following sampling for previously mentioned outcomes and after gross imaging, devices were fixed in 4% paraformaldehyde and processed for histological evaluation. Devices were dehydrated, infiltrated with Technovit 7100 resin, and embedded in methacrylate plastic. Longitudinal sections, 5-µm thick, were cut from each device with a Leica microtome and stained with Richard-Allan Scientific hematoxylin and eosin (Richard-Allan Scientific, Kalamazoo, MI, USA). Stained sections were evaluated using light microscopy images at 40 × and 100 × magnification by 3 independent observers who determined the cell density, and cell morphology on a scale of 0 to 3, with 3 indicating normal cell morphology, with little or no signs of cell death and excellent cell density (Supplementary Table S2, Supplementary Fig. S1).

### Ethical Considerations

All trials were conducted in full compliance with the Declaration of Helsinki and protocols were approved at all sites by the local or central institutional review board. All participants provided written informed consent.

### Statistical Analysis

Descriptive statistics of inter- and intra-time point and group data for CNTF release levels and specific activity were performed using Prism Software version 10.0.2 (GraphPad, Boston, MA, USA)*.* Weighted average (geometric) means were calculated in all sample time points. Geometric mean better reflects this dataset in the current design because of unequal populations across implant duration, where a small sample size in one element may shift the result.

## Results

### Sample Distribution

A total of 49 explanted revakinagene taroretcel-lwey devices from six clinical trials and one compassionate use protocol were examined for this retrospective analysis. A list of the participants, their respective clinical trials, and the duration of implantation is presented in Supplementary Table S1.

### CNTF Pharmacokinetics From Explanted Devices

**Figure 2.**
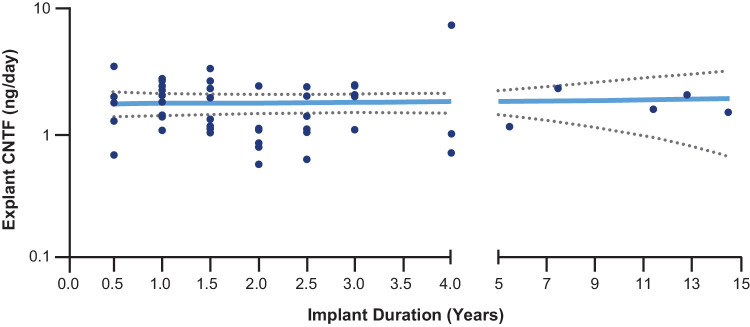
Ciliary neurotrophic factor (CNTF) pharmacokinetics from explanted revakinagene taroretcel-lwey devices. Secretion of CNTF (ng/day) by revakinagene taroretcel-lwey devices explanted over 14.5 years of implant durations, grouped to the nearest half-year for durations <3 years. The *solid line* indicates the weighted mean CNTF output level, and the *dotted lines* indicate the range of the 95% confidence interval of the slope. CNTF, ciliary neurotrophic factor; ng, nanogram.

For the 49 devices that were explanted between 6 months and 14.5 years, the geometric mean of released CNTF was 1.598 ng/day (95% confidence interval [CI] = 1.39 to 1.84). The slope of CNTF release from explanted devices was 0.0137 (95% CI = −0.09 to 0.12) over 14.5 years, which is not significantly different than zero (Fig. 2). Data from multiple explanted devices demonstrate that the duration of CNTF release exceeds 10 years (Supplementary Table S3). All of the explants were found to release CNTF.

### Evaluation of CNTF Bioactivity

**Figure 3.**
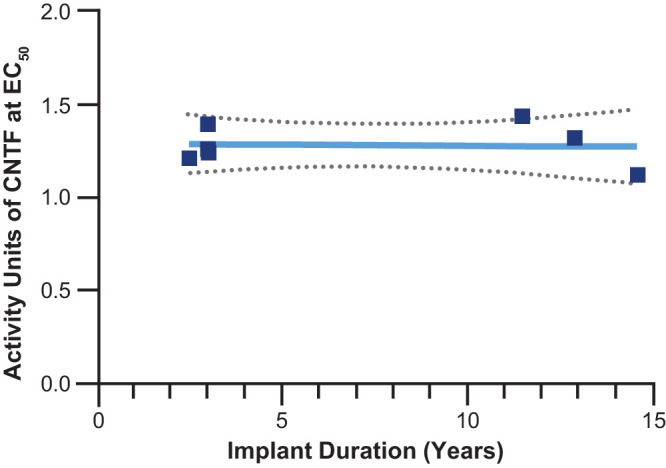
Units of activity of CNTF produced by revakinagene taroretcel-lwey explants over 14.5 years of implant duration. Seven samples were analyzed, 2 of which were analyzed twice, and the results were averaged. The *solid line* indicates the slope of units of activity, and the *dotted lines* indicate the range of the 95% confidence interval of the slope. CNTF, ciliary neurotrophic factor; EC_50_, half-maximal effective concentration.

Revakinagene taroretcel-lwey explant release of CNTF was tested for bioactivity relative to a reference CNTF. Specific activity of the samples averaged 1.26 activity units (95% CI = 1.19 to 1.33) over that of the reference CNTF and, given the variability of the assay, were not significantly different from each other over the duration of the implant period (slope = 0.0058; 95% CI = −0.02 to 0.01; Fig. 3). The activity of the secreted CNTF over the duration of the implant remained equally bioactive compared with that produced by the reference CNTF (Supplementary Table S4).

### Evaluation of CNTF Identity

**Figure 4.**
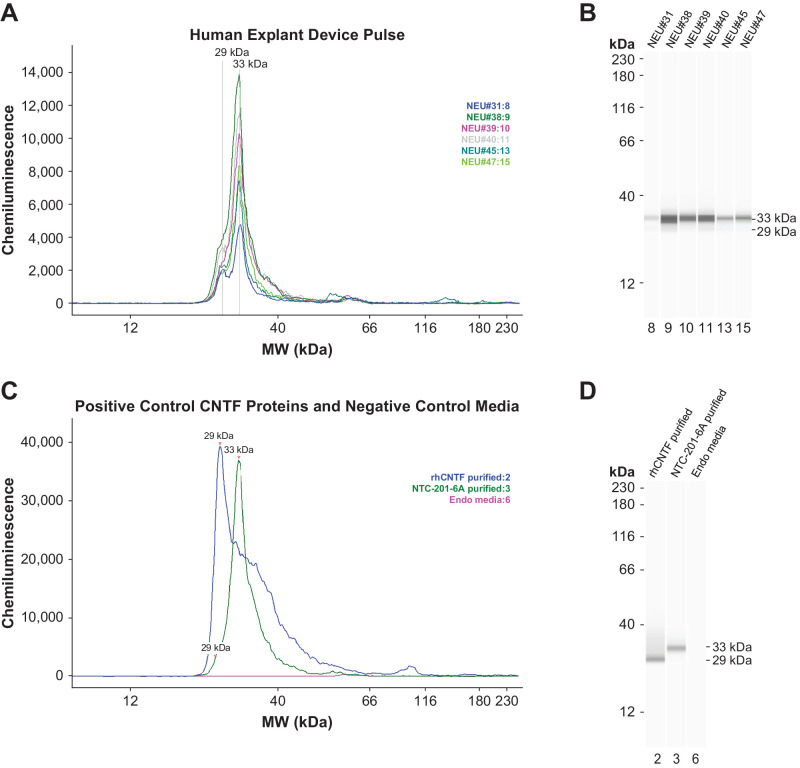
Electropherograms of CNTF from explanted revakinagene taroretcel-lwey. (**A**) Electropherograms and (**B**) corresponding bands from CNTF-conditioned media collected from devices explanted from 6 participants over 14.5 years. The explant-derived CNTF is a mixture of 29 kDa human CNTF and the 33 kDa glycosylated CNTF unique to NTC-201-6A cells. Electropherograms of (**C**) controls and (**D**) corresponding bands are shown. The controls used in this analysis include purified CNTF produced in bacteria, purified revakinagene taroretcel-lwey–secreted CNTF, and culture media. Purified CNTF protein produced in bacteria consistently has a high MW shoulder. CNTF, ciliary neurotrophic factor; kDa, kilodalton; MW, molecular weight.

Revakinagene taroretcel-lwey explant release of CNTF was analyzed for identity by capillary-based immunoassay in comparison to a molecular weight ladder, an rhCNTF reference protein, and CNTF protein purified from NTC-201-6A cells. CNTF released by revakinagene taroretcel-lwey device explants conformed to the molecular weight of the expected glycoforms found only in NTC-201-6A cells. CNTF produced by NTC-201-6A is O-linked glycosylated at the N-terminus of the protein resulting in a higher-than-expected molecular weight peak along with the expected size of non-glycosylated native CNTF peak. Deglycosylation of revakinagene taroretcel-lwey CNTF reduces the larger peak to the size of non-glycosylated CNTF. Both glycoforms of purified CNTF are equally bioactive in the TF1 bioassay (Nystuen AM, et al. *Invest Ophthalmol Vis Sci* 2025;66. ARVO E-Abstract 5943). All explanted devices, regardless of implant duration, produced similar electropherograms (Figs. 4A, 4B). Recombinant CNTF, purified NTC-201-6A CNTF, and a media background negative control are shown in Figures 4C and 4D.

### CNTF Serum Levels and Immunologic Response

Preimplant and postimplant serum samples were collected from participants according to the clinical trial protocols. These serum samples were analyzed by ELISA for CNTF levels in ng/mL of serum. All samples were below the lower limit of quantification (data not shown). Serum samples were also screened for antibodies against CNTF and antibodies against the NTC-201-6A cell line. No increase in antibody titer was identified in any participant over their baseline sample (data not shown).

### Macroscopic Evaluation of Revakinagene Taroretcel-lwey Explants

**Figure 5.**
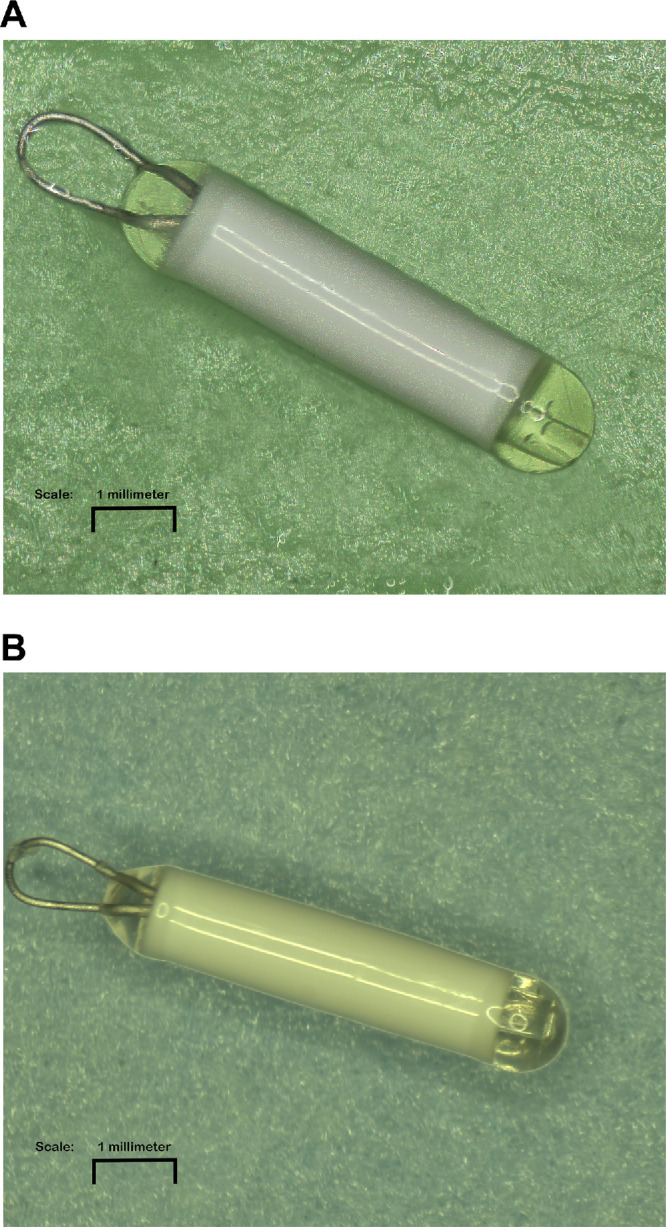
Macroscopic images of (**A**) a preimplant device as a reference and (**B**) an explanted revakinagene taroretcel-lwey after implant duration of 14.5 years. The image demonstrates no evidence of gross damage or tissue adhered to the white membrane. Image is magnified 25 ×.

Upon macroscopic examination of the revakinagene taroretcel-lwey explants, including the longest duration 14.5-year explant, there was no visible discoloration, tissue adherence to the membrane, or obvious material breakdown observed. Gross observational results were consistent across all explants. An image of a preimplant reference device and a 14.5-year explanted device each at a magnification of 25 × is shown in Figure 5.

### Histological Evaluation of Revakinagene Taroretcel-lwey Explants

Explanted devices were histologically evaluated for cell density and gross cellular morphology. Images of explanted device sections were taken at 40 × and 100 × magnification and independently graded by 3 analysts. The scores representing the cell morphology at 100 × and device cell density at 40 × are shown in Supplementary Table S5. The morphology and cell density of the majority of explanted devices remained relatively constant over all device implant durations among all 3 analysts (see Supplementary Table S5). The device with an implant duration of 14.5 years showed no dramatic loss of cell density, and most cells were morphologically normal.

**Figure 6.**
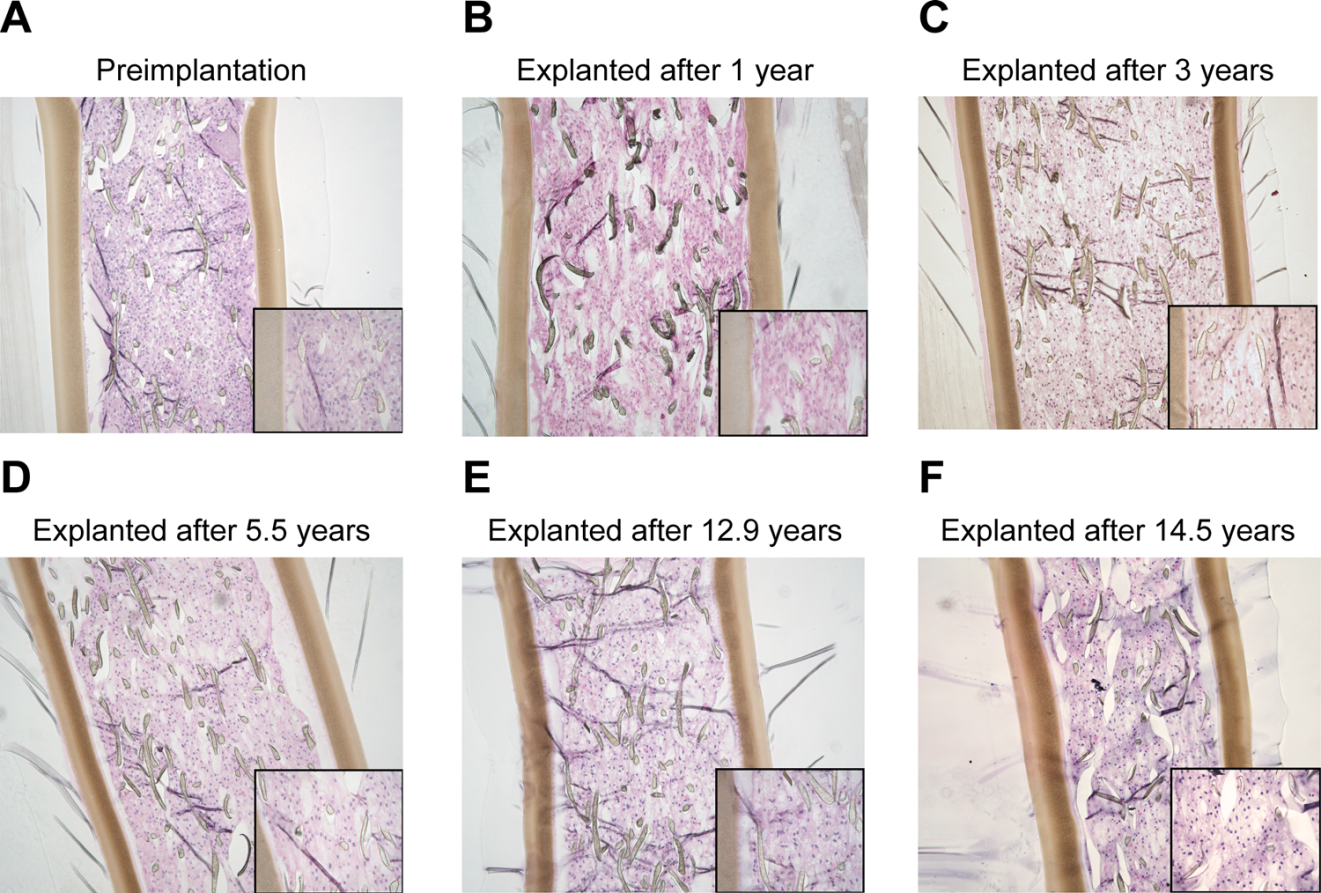
Microscopic images of (**A**) a preimplant device as a reference and (**B–F**) explanted devices from various durations of implantation. (**A–E**) Representative hematoxylin and eosin–stained sections of preimplant encapsulated NTC-201-6A cells and encapsulated cells from revakinagene taroretcel-lwey explants over 14.5 years.

In general, the cells from each of the 48 explants across the 6 clinical trials revealed similar cell size, morphology, and population distributions. Morphologically, the nuclei of the explanted cells were round or oval with some pleomorphism. The presence of nucleoli, nuclear grooves, and focal multinucleation was noted, as was the absence of mitotic figures. There was evidence of cells present with intercellular and intracellular cysts with apparent fluid, vacuolization, and inspissated pink material (potentially basement membrane debris). Figure 6 displays representative images at 100 × magnification, each with an insert image at 400 × magnification. These images are from explanted devices with various implant durations, alongside a preimplant reference image.

## Discussion

This retrospective analysis of explanted devices from 6 clinical trials demonstrated that revakinagene taroretcel-lwey released consistent levels of bioactive CNTF over an extended duration of up to 14.5 years, confirming its durability in long-term therapeutic applications. The rate of CNTF release of 1.6 ng/day has been shown to be effective in slowing photoreceptor degeneration both in animal models and clinical studies.^7^^,^^16^ In the *rcd-1* mutant canine model of RP, dose-dependent photoreceptor protection was observed with CNTF release from revakinagene taroretcel-lwey of 0 ng/day (parental/non-transfected cell line that does not release detectable levels of CNTF) through 15 ng/day, with therapeutic benefit demonstrated above 0.2 ng/day.^16^ Similarly, in clinical trials conducted in participants with MacTel, the rate of CNTF secretion from revakinagene taroretcel-lwey was sufficient to slow photoreceptor loss as evidenced by a significant reduction in the rate of ellipsoid zone area loss through 2 years compared with sham treatment in one phase II and two phase III clinical trials.^7^^,^^17^ The consistent rate of CNTF release for over 14 years shown here is a promising indicator of the potential role that revakinagene taroretcel-lwey may play as a therapeutic option in chronic retinal diseases.

The current retrospective analysis builds on the previously reported 2-year data by Kauper et al.,^14^ in which it was reported that revakinagene taroretcel-lwey released stable CNTF at rates similar to those reported here over a 2-year period. This analysis extends the duration of treatment to more than 14 years, demonstrating the longevity of revakinagene taroretcel-lwey in delivering bioactive CNTF; through bioactivity assays, this analysis confirmed that the CNTF released from the explants remained functional over time, reinforcing that ECT can provide sustained, consistent therapeutic release in the setting of chronic retinal degenerative disease.

In this analysis, all revakinagene taroretcel-lwey explants were found to release CNTF. Additionally, histological evaluation determined that cells from revakinagene taroretcel-lwey explants were similar in density and morphology, demonstrating that cell viability and function of revakinagene taroretcel-lwey is preserved in the human vitreous throughout the extended implantation period. Together, these analyses underscore that revakinagene taroretcel-lwey can provide sustained therapeutic effects over a long duration. The lack of a fibrotic foreign body response and the relative immune privilege make the eye a uniquely suited environment for the use of encapsulated cells to deliver protein therapeutics, although extended systemic delivery of biologics from devices implanted subcutaneously in mice has been demonstrated.^22^ This supports the possibility of ECT as a flexible platform that could potentially be used for localized therapeutic delivery in other chronic diseases.

This retrospective analysis had limitations, including the small sample size in bioactivity and identity analysis. Additionally, there was an unequal distribution of samples across implant duration due to a majority of implanted participants choosing to retain their implant at the conclusion of their respective studies. This may affect the generalizability of these findings. A further limitation was that histological analysis of devices necessitated the use of a plastic resin that is incompatible with immunostaining; therefore, the presence of host immune components within explanted devices cannot be determined. Although this is one of the few studies examining the long-term durability of ECT, future studies assaying aqueous levels of CNTF over time would further validate these findings and could establish revakinagene taroretcel-lwey as a viable long-term option for managing chronic retinal degenerative diseases.

These findings indicate that revakinagene taroretcel-lwey, surgically inserted into the vitreous, delivers sustained bioactive CNTF for more than a decade, potentially providing long-term treatment for chronic retinal degenerative diseases.

## Supplementary Material

Supplement 1
