# Supplementary material for: Long-Term Durability of Ciliary Neurotrophic Factor–Releasing Revakinagene Taroretcel-lwey in Individuals With Retinal Degenerative Disorders
Source: Invest Ophthalmol Vis Sci. 2025 Aug 4;66(11):3. doi: 10.1167/iovs.66.11.3 (PMC12327535; doi:10.1167/iovs.66.11.3)
Supplement: Supplement 1 [file iovs-66-11-3_s001.docx]

**Supplementary Materials**

**Supplementary Table 1.** Summary of device explant samples.

| **Sample ID** | **Trial Number** | **Implant Date** | **Explant Date** | **Duration (Years)** |
| --- | --- | --- | --- | --- |
| NEU#1 | NCT00063765 | 2004-10-25 | 2005-04-01 | 0.43 |
| NEU#2 | NCT00063765 | 2004-10-25 | 2005-04-01 | 0.43 |
| NEU#3 | NCT00063765 | 2004-07-20 | 2005-01-01 | 0.45 |
| NEU#4 | NCT00063765 | 2005-09-20 | 2005-03-01 | 0.45 |
| NEU#5 | NCT00063765 | 2004-06-07 | 2004-12-01 | 0.48 |
| NEU#6 | NCT00447993 | 2007-10-05 | 2008-09-10 | 0.93 |
| NEU#7 | NCT00447993 | 2007-04-23 | 2008-04-21 | 0.99 |
| NEU#8 | NCT00447993 | 2007-09-20 | 2008-09-23 | 1.01 |
| NEU#9 | NCT00447993 | 2007-04-20 | 2008-05-07 | 1.05 |
| NEU#10 | NCT00447993 | 2007-10-09 | 2008-11-06 | 1.08 |
| NEU#11 | NCT00447954 | 2007-11-02 | 2008-12-05 | 1.09 |
| NEU#12 | NCT00447993 | 2007-04-30 | 2008-06-09 | 1.11 |
| NEU#13 | NCT00447993 | 2007-07-06 | 2008-08-15 | 1.11 |
| NEU#14 | NCT00447993 | 2007-09-27 | 2008-11-13 | 1.13 |
| NEU#15 | NCT00447993 | 2007-10-24 | 2009-01-13 | 1.22 |
| NEU#16 | NCT00447980 | 2007-07-30 | 2008-11-12 | 1.28 |
| NEU#17 | NCT00447954 | 2007-09-18 | 2009-01-16 | 1.33 |
| NEU#18 | NCT00447993 | 2007-08-02 | 2009-01-08 | 1.43 |
| NEU#19 | NCT00447954 | 2007-08-02 | 2009-02-05 | 1.51 |
| NEU#20 | NCT00447993 | 2007-08-15 | 2009-02-18 | 1.51 |
| NEU#21 | NCT00447993 | 2007-04-24 | 2008-11-18 | 1.57 |
| NEU#22 | NCT00447993 | 2007-04-24 | 2008-11-18 | 1.57 |
| NEU#23 | NCT00447993 | 2007-02-14 | 2008-09-10 | 1.57 |
| NEU#24 | NCT00447980 | 2007-06-20 | 2009-06-17 | 1.99 |
| NEU#25 | NCT00447980 | 2007-06-13 | 2009-06-17 | 2.01 |
| NEU#26 | NCT00447980 | 2007-08-02 | 2009-08-11 | 2.03 |
| NEU#27 | NCT00447980 | 2007-06-07 | 2009-07-01 | 2.07 |
| NEU#28 | NCT00447980 | 2007-04-25 | 2009-05-20 | 2.07 |
| NEU#29 | NCT00447980 | 2007-06-28 | 2009-08-11 | 2.12 |
| NEU#30 | NCT00447980 | 2007-06-06 | 2009-11-10 | 2.43 |
| NEU#31 | NCT01530659 | 2016-02-09 | 2018-07-31 | 2.48 |
| NEU#32 | NCT00447980 | 2007-05-22 | 2009-11-17 | 2.49 |
| NEU#33 | NCT00447980 | 2007-06-06 | 2009-12-09 | 2.51 |
| NEU#34 | NCT00447980 | 2007-02-14 | 2009-09-02 | 2.55 |
| NEU#35 | NCT00447980 | 2007-04-25 | 2009-12-09 | 2.62 |
| NEU#36 | NCT01530659 | 2016-02-09 | 2019-01-15 | 2.93 |
| NEU#37 | NCT01530659 | 2016-02-23 | 2019-02-12 | 2.97 |
| NEU#38 | NCT01530659 | 2016-03-08 | 2019-03-19 | 3.03 |
| NEU#39 | NCT01530659 | 2016-03-01 | 2019-03-19 | 3.05 |
| NEU#40 | NCT01530659 | 2016-03-01 | 2019-03-26 | 3.07 |
| NEU#41 | NCT01530659 | 2016-02-23 | 2019-05-28 | 3.26 |
| NEU#42 | NCT00447980 | 2007-07-06 | 2011-03-03 | 3.66 |
| NEU#43 | NCT03319849 | 2019-07-11 | 2023-07-12 | 4.00 |
| NEU#44 | NCT00447980 | 2007-11-02 | 2012-05-20 | 4.55 |
| NEU#45 | NCT00447980 | 2007-06-01 | 2012-11-20 | 5.47 |
| NEU#46 | Emory compassionate Protocol Emory 201-CU01-RDD-2011 | 2011-08-17 | 2019-02-12 | 7.49 |
| NEU#47 | NCT00447993 | 2007-06-26 | 2018-11-27 | 11.42 |
| NEU#48 | NCT00447993 | 2007-07-24 | 2020-05-19 | 12.82 |
| NEU#49 | NCT00447993 | 2007-11-14 | 2022-05-20 | 14.52 |

**Supplementary Table 2.** Cell morphology and density rating system.

| **Cell Morphology Within an Explanted Device** | |
| --- | --- |
| **Rating** | **Description** |
| **0** | No viable cells observed, no nuclei visible |
| **1** | Majority unhealthy/dying cells, >75% |
| **2** | A mix of healthy and unhealthy cells visible |
| **3** | Majority cells are viable >75% with normal morphology; minority of individual cells may show abnormal morphology or undergoing apoptosis |
| **Cell Density Within an Explanted Device** | |
| **Rating** | **Description** |
| **0** | No cells observed |
| **1** | Poor cell density <25% occupied volume |
| **2** | Moderate cell density; 25–75% occupied volume |
| **3** | Good cell density; ≥75% occupied volume |

**Supplementary Table 3.** CNTF pharmacokinetics from explanted devices.

| **Sample ID** | **Implant Duration (Years)** | **Grouped Time Point (Years)** | **CNTF Release (ng/day)** | **CNTF Weighted Average Grouped Time Point (ng/day)** |
| --- | --- | --- | --- | --- |
| NEU#1 | 0.43 | 0.5 | 1.313 | 1.53 |
| NEU#2 | 0.43 |  | 2.032 |  |
| NEU#3 | 0.45 |  | 1.846 |  |
| NEU#4 | 0.45 |  | 1.786 |  |
| NEU#5 | 0.48 |  | 0.684 |  |
| NEU#6 | 0.93 | 1.0 | 2.784 | 2.01 |
| NEU#7 | 0.99 |  | 2.264 |  |
| NEU#8 | 1.01 |  | 1.087 |  |
| NEU#9 | 1.05 |  | 1.823 |  |
| NEU#10 | 1.08 |  | 2.077 |  |
| NEU#11 | 1.09 |  | 2.044 |  |
| NEU#12 | 1.11 |  | 2.448 |  |
| NEU#13 | 1.11 |  | 1.436 |  |
| NEU#14 | 1.13 |  | 1.404 |  |
| NEU#15 | 1.22 |  | 2.71 |  |
| NEU#16 | 1.28 | 1.5 | 2.693* | 1.88 |
| NEU#17 | 1.33 |  | 1.177 |  |
| NEU#18 | 1.43 |  | 2.346 |  |
| NEU#19 | 1.51 |  | 1.337 |  |
| NEU#20 | 1.51 |  | 1.038 |  |
| NEU#21 | 1.57 |  | 1.126* |  |
| NEU#22 | 1.57 |  | 1.981* |  |
| NEU#23 | 1.57 |  | 3.376 |  |
| NEU#24 | 1.99 | 2.0 | 2.453 | 1.15 |
| NEU#25 | 2.01 |  | 0.798 |  |
| NEU#26 | 2.03 |  | 0.863 |  |
| NEU#27 | 2.07 |  | 1.12 |  |
| NEU#28 | 2.07 |  | 0.581 |  |
| NEU#29 | 2.12 |  | 1.101 |  |
| NEU#30 | 2.43 | 2.5 | 1.054 | 1.45 |
| NEU#31 | 2.48 |  | 2.049 |  |
| NEU#32 | 2.49 |  | 1.415 |  |
| NEU#33 | 2.51 |  | 0.639 |  |
| NEU#34 | 2.55 |  | 1.108 |  |
| NEU#35 | 2.62 |  | 2.414 |  |
| NEU#36 | 2.93 | 3.0 | 2.086 | 2.04 |
| NEU#37 | 2.97 |  | 1.098 |  |
| NEU#38 | 3.03 |  | 2.495 |  |
| NEU#39 | 3.05 |  | 2.049 |  |
| NEU#40 | 3.07 |  | 2.473 |  |
| NEU#41 | 3.26 |  | 2.041 |  |
| NEU#42 | 3.66 | 4 | 1.019 | 3.11 |
| NEU#43 | 4.00 |  | 7.6 |  |
| NEU#44 | 4.55 |  | 0.717 |  |
| NEU#45 | 5.47 | 5.47 | 1.162 | 1.16 |
| NEU#46 | 7.49 | 7.49 | 2.345 | 2.35 |
| NEU#47 | 11.42 | 11.42 | 1.602 | 1.6 |
| NEU#48 | 12.82 | 12.82 | 2.08 | 2.08 |
| NEU#49 | 14.52 | 14.52 | 1.508 | 1.51 |

Device explant pulses were analyzed for CNTF levels by ELISA. Implant duration is given in years, rounded to the neared half-year for durations <3 years.

CNTF, ciliary neurotrophic factor; ELISA, enzyme-linked immunosorbent assay.

**Supplementary Table 4.** Units of activity of CNTF at EC_50_ from explanted devices compared with reference CNTF, with samples analyzed in triplicate.

| **Sample ID** | **Implant Duration (Years)** | **Explant Values at EC_50_ (OD)** | **Explant Mean Value (OD)** | **Reference Protein Values at EC_50_ (OD)** | **Reference Protein Mean Value (OD)** | **Units of Activity Explant/**  **Reference** |  |
| --- | --- | --- | --- | --- | --- | --- | --- |
|  |  |  |  |  |  |  |  |
| NEU#48 | 12.82 | 1.696 | 1.548 | 1.057 | 1.186 | 1.305 |  |
|  |  | 1.536 |  | 1.263 |  |  |  |
|  |  | 1.412 |  | 1.237 |  |  |  |
| NEU#47^a^ | 11.42 | 1.835 | 1.793 | 1.057 | 1.186 | 1.512 |  |
|  |  | 1.702 |  | 1.263 |  |  |  |
|  |  | 1.843 |  | 1.237 |  |  |  |
| NEU#31 | 2.48 | 1.536 | 1.514 | 1.057 | 1.186 | 1.277 |  |
|  |  | 1.508 |  | 1.263 |  |  |  |
|  |  | 1.498 |  | 1.237 |  |  |  |
| NEU#38* | 3.03 | 1.751 | 1.692 | 1.057 | 1.186 | 1.427 |  |
|  |  | 1.639 |  | 1.263 |  |  |  |
|  |  | 1.685 |  | 1.237 |  |  |  |
| NEU#40 | 3.05 | 1.61 | 1.517 | 1.057 | 1.186 | 1.279 |  |
|  |  | 1.475 |  | 1.263 |  |  |  |
|  |  | 1.467 |  | 1.237 |  |  |  |
| NEU#39 | 3.07 | 1.999 | 2.027 | 1.537 | 1.61 | 1.259 |  |
|  |  | 2.074 |  | 1.618 |  |  |  |
|  |  | 2.007 |  | 1.674 |  |  |  |
| NEU#38^*,†^ | 3.03 | 2.33 | 2.320 | 2.126 | 2.096 | 1.107 |  |
|  |  | 2.3 |  | 2.002 |  |  |  |
|  |  | 2.445 |  | 2.228 |  |  |  |
|  |  | 2.375 |  | 2.115 |  |  |  |
|  |  | 2.322 |  | 1.813 |  |  |  |
|  |  | 2.146 |  | 2.293 |  |  |  |
| NEU#47^†^ | 11.42 | 2.143 | 2.302 | 2.126 | 2.096 | 1.098 |  |
|  |  | 2.376 |  | 2.002 |  |  |  |
|  |  | 2.304 |  | 2.228 |  |  |  |
|  |  | 2.211 |  | 2.115 |  |  |  |
|  |  | 2.43 |  | 1.813 |  |  |  |
|  |  | 2.35 |  | 2.293 |  |  |  |
| NEU#49^†^ | 14.52 | 2.35 | 2.286 | 2.126 | 2.096 | 1.091 |  |
|  |  | 2.14 |  | 2.002 |  |  |  |
|  |  | 2.351 |  | 2.228 |  |  |  |
|  |  | 2.55 |  | 2.115 |  |  |  |
|  |  | 2.177 |  | 1.813 |  |  |  |
|  |  | 2.148 |  | 2.293 |  |  |  |

Within an assay group, the reference protein OD values and means were from the same replicates.

*Samples NEU#38 and NEU#47 were evaluated on two assays; in the final data the average units of CNTF activities were 1.267 and 1.305, respectively.

^†^Three samples had adequate sample volume to perform six independent dilutions.

CNTF, ciliary neurotrophic factor; EC_50_, half-maximum effective concentration; ng/mL, nanograms per milliliter; OD, optical density.

**Supplementary Table 5.** Histology score of explanted devices.

| **Study ID** | **Grouped Implant Duration (years)^*^** | **Cell Morphology/Health/Viability  Within an Explanted Device^†^** | | | **Cell Density Within an  Explanted Device^†^** | | |
| --- | --- | --- | --- | --- | --- | --- | --- |
|  |  | **Analyst 1** | **Analyst 2** | **Analyst 3** | **Analyst 1** | **Analyst 2** | **Analyst 3** |
| NEU#1 | 0.5 | 2 | 2 | 2 | 3 | 1 | 2 |
| NEU#2 |  | 2 | 1 | 2 | 3 | 2 | 2 |
| NEU#3 |  | 3 | 3 | 3 | 3 | 2 | 3 |
| NEU#4 |  | 3 | 3 | 3 | 3 | 2 | 3 |
| NEU#5 |  | 3 | 3 | 3 | 3 | 3 | 3 |
| NEU#6 | 1.0 | 2 | 2 | 2 | 2 | 1 | 1 |
| NEU#7 |  | 3 | 3 | 3 | 3 | 3 | 3 |
| NEU#8 |  | 2 | 3 | 3 | 3 | 2 | 3 |
| NEU#9 |  | 3 | 3 | 3 | 3 | 3 | 3 |
| NEU#10^‡^ |  | 1 | 1 | 1 | 1 | 1 | 2 |
| NEU#11 |  | 3 | 3 | 3 | 3 | 3 | 3 |
| NEU#12 |  | 3 | 3 | 3 | 3 | 3 | 3 |
| NEU#13 |  | 2 | 3 | 2 | 3 | 3 | 3 |
| NEU#14 |  | 3 | 3 | 3 | 3 | 2 | 3 |
| NEU#15 |  | 3 | 3 | 3 | 2 | 2 | 2 |
| NEU#16 | 1.5 | 3 | 3 | 3 | 3 | 3 | 3 |
| NEU#17 |  | 3 | 3 | 3 | 2 | 3 | 2 |
| NEU#18 |  | 3 | 3 | 3 | 3 | 3 | 3 |
| NEU#19 |  | 3 | 3 | 3 | 3 | 3 | 3 |
| NEU#20 |  | 3 | 3 | 3 | 3 | 2 | 3 |
| NEU#21 |  | 3 | 3 | 3 | 3 | 3 | 3 |
| NEU#22 |  | 3 | 3 | 3 | 3 | 3 | 3 |
| NEU#23 |  | 1 | 2 | 2 | 2 | 1 | 1 |
| NEU#24 | 2.0 | 2 | 2 | 2 | 2 | 2 | 3 |
| NEU#25 |  | 3 | 3 | 2 | 3 | 3 | 3 |
| NEU#26 |  | 3 | 3 | 3 | 3 | 3 | 3 |
| NEU#27 |  | 3 | 3 | 3 | 3 | 3 | 3 |
| NEU#28 |  | 2 | 3 | 2 | 3 | 3 | 3 |
| NEU#29 |  | 3 | 3 | 3 | 2 | 2 | 3 |
| NEU#30 | 2.5 | 3 | 3 | 3 | 3 | 3 | 3 |
| NEU#31 |  | 3 | 3 | 3 | 3 | 3 | 3 |
| NEU#32 |  | 3 | 3 | 3 | 3 | 3 | 3 |
| NEU#33 |  | 2 | 3 | 3 | 3 | 2 | 2 |
| NEU#34 |  | 3 | 3 | 3 | 3 | 3 | 3 |
| NEU#35 |  | 3 | 3 | 3 | 3 | 3 | 3 |
| NEU#36 | 3.0 | 3 | 3 | 3 | 3 | 3 | 3 |
| NEU#37 |  | 3 | 3 | 3 | 3 | 3 | 3 |
| NEU#38 |  | 3 | 3 | 3 | 3 | 3 | 3 |
| NEU#39 |  | 3 | 3 | 3 | 3 | 3 | 3 |
| NEU#40 |  | 3 | 3 | 3 | 3 | 3 | 3 |
| NEU#41 |  | Sample not available for histological analysis | | | | | |
| NEU#42 | 4 | 3 | 3 | 3 | 3 | 3 | 3 |
| NEU#43 |  | 3 | 3 | 3 | 3 | 3 | 3 |
| NEU#44 |  | 3 | 3 | 3 | 3 | 3 | 3 |
| NEU#45 | 5.47 | 3 | 3 | 3 | 3 | 3 | 3 |
| NEU#46 | 7.49 | 3 | 3 | 3 | 3 | 3 | 3 |
| NEU#47 | 11.42 | 1 | 3 | 2 | 2 | 2 | 2 |
| NEU#48 | 12.82 | 3 | 3 | 3 | 3 | 3 | 3 |
| NEU#49 | 14.52 | 3 | 3 | 3 | 3 | 2 | 3 |

*Grouped implant duration is rounded to the nearest half year for durations ≤3 years.

^†^Cell morphology and density were evaluated and scored from 0 to 3 by three different analysts.

^‡^Factors that may negatively influence the appearance of the explant include handling or shipment conditions of the explant prior to histologic processing and potential for human error in histopathological processing. While NEU#10 had lower histological scores, the device released 2.077 ng/day of CNTF following explantation, which is within the CNTF range of the other 48 samples. See **Supplementary Table 3** for individual device explant levels.

**Supplementary Figure 1.** Macroscopic image of histology-scored explanted devices. (**A**) An explant of revakinagene taroretcel-lwey rated 1 for both cell morphology and cell density and (**B**) an explant rated 3 for both cell morphology and cell density.

**A**

**
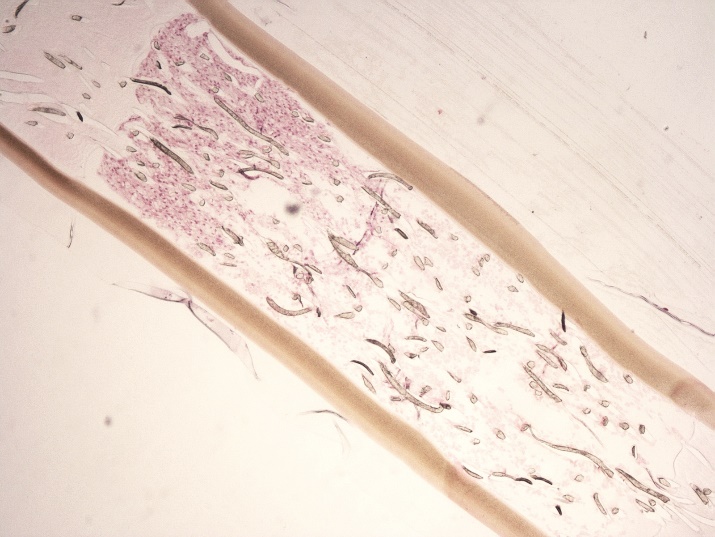
**

**B**

**
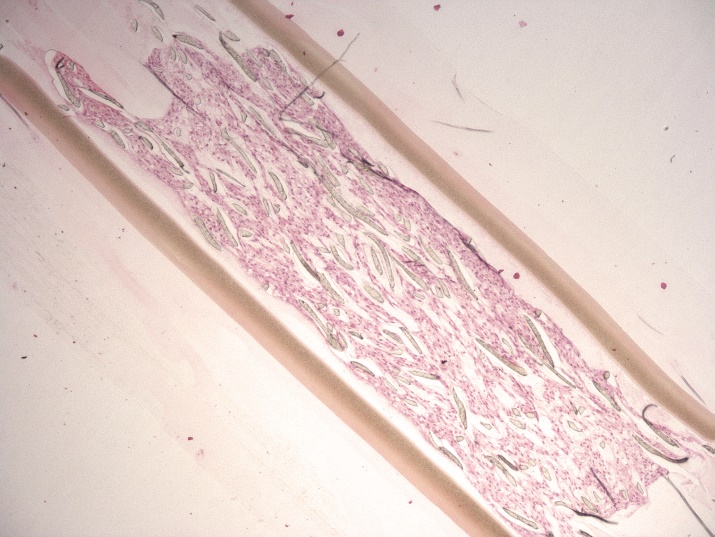
**
